# Anti-HER2-targeted therapies: effects on human *in vitro* blood-brain barrier models

**DOI:** 10.3389/fddev.2025.1700455

**Published:** 2026-01-07

**Authors:** Carolin J. Curtaz, Rebecca Gebert, Achim Wöckel, Patrick Meybohm, Malgorzata Burek

**Affiliations:** 1 Department of Gynecology and Obstetrics, University Hospital Würzburg, Würzburg, Germany; 2 Department of Anaesthesiology, Intensive Care, Emergency and Pain Medicine, University Hospital Würzburg, Würzburg, Germany

**Keywords:** BLECs, brain microvascular endothelial cells, hCMEC/D3, HER2-positive breast cancer, lapatinib, pertuzumab, trastuzumab, tucatinib

## Abstract

Metastatic breast cancer is associated with very poor overall survival and a reduced quality of life. HER2-positive breast cancer forms brain metastases at the late stages. Established therapies such as trastuzumab, pertuzumab, trastuzumab/pertuzumab, lapatinib and tucatinib are widely used and are selectively toxic to HER2-positive breast cancer cell line. However, the effects of these therapies on the properties of the blood-brain barrier (BBB) remain unclear. We investigated this using an *in vitro* human BBB model derived from CD34^+^ cells differentiated into brain-like endothelial cells (BLECs) and hCMEC/D3 cell line. BLECs were treated with different concentrations of trastuzumab, pertuzumab, trastuzumab/pertuzumab, lapatinib or tucatinib for 24 h and 48 h. We measured cell viability, transendothelial electrical resistance (TEER), paracellular permeability to fluorescein and mRNA expression profiles. Most treatments showed no effect on cell viability, permeability and TEER of endothelial cells. While treatment of BLECs with lapatinib and tucatinib at low concentrations resulted in increased cell viability/metabolism, treatment with a higher concentration of 5 μg/mL resulted in toxic effects. These results were confirmed using another BBB *in vitro* model, hCMEC/D3. Treatment with trastuzumab and trastuzumab/pertuzumab resulted in changes in the mRNA expression of BBB marker genes encoding efflux pumps (P-gp (ABCB1)/BCRP (ABCG2)), the glucose transporter GLUT-1 (SLC2A1), tight junction proteins (occludin (OCLN)/claudin-5 (CLDN5)) and the pro-inflammatory chemokine CCL2. In conclusion, we demonstrate different time- and concentration-dependent effects of anti-HER2-targeted therapies for the treatment of advanced HER2-positive breast cancer on the BBB *in vitro*. Further experiments are required to assess the clinical relevance of our results.

## Introduction

In about 20% of all breast cancers there is an amplification of human epidermal growth factor receptor 2 (HER2). This breast cancer subtype is particularly aggressive and leads to distant metastases including brain metastases. Due to increasingly effective treatment options for metastatic breast cancer and the long disease progression in the metastatic stage, a growing number of patients are suffering from cerebral metastases, especially in the HER2-positive breast cancer subgroup (Müller et al., 2025). Current therapies for the treatment of HER2-positive metastatic breast cancer include the anti-HER2 monoclonal antibodies trastuzumab and pertuzumab. HER2 signaling can be blocked by the binding of antibodies to the HER2 receptor on the surface of cancer cells resulting in reduced cell growth. Another way to block HER2 signaling is to use small tyrosine kinase inhibitors (TKI), such as lapatinib and tucatinib, which act at the intracellular domain of the HER2 receptor and block its autophosphorylation and signal transduction (Geyer et al., 2006; Lin et al., 2023). Due to their small size, TKI show better tissue penetration and lead to promising patient outcomes in clinical trials (Gril et al., 2008; Lin et al., 2020). Overall, the clinical treatment of cerebral metastases in breast cancer patients presents a challenge. Clinical trials on the efficacy of drugs in HER2-positive breast cancer with brain metastases have only recently been conducted and primarily demonstrate the benefit of tyrosine kinase inhibitors and novel drug conjugates (Müller et al., 2025). However, it remains unclear how the commonly used anti-HER2 drugs for HER2-positive metastatic breast cancer reach the brain metastases across the blood-brain barrier (BBB).

BBB, composed of brain microvascular endothelial cells, pericytes, a basement membrane and astrocytes, maintains brain homeostasis. Tight junctions between the neighboring endothelial cells, together with transporter molecules regulate and maintain the barrier properties of the BBB. When the BBB is compromised by certain factors, its barrier properties are impaired, which, among other things, means that tumor cells can more easily metastasize to the brain. Expression changes of BBB marker genes and proteins, such as claudin-5, occludin, efflux pumps and glucose transporter contribute to dysfunction of the BBB in various diseases.

*In vitro* models are a useful tool for analyzing BBB properties (Helms et al., 2016). In the present study, we used human brain-like endothelial cells (BLECs) derived from CD34^+^ cells in co-culture with brain pericytes and an immortalized brain microvascular endothelial hCMEC/D3 cell line as *in vitro* BBB models (Cecchelli et al., 2014; Helms et al., 2016; Curtaz et al., 2020).

The *in vitro* BBB model derived from CD34^+^ cells was first published by Cecchelli et al., in 2014 (Cecchelli et al., 2014). In our previous studies using this *in vitro* model, we observed the induction of tight junction proteins, transporters and cellular receptors at the protein or mRNA level when the cells were co-cultured with brain pericytes (Curtaz et al., 2020). Pericytes induce BBB properties in CD34^+^ derived hematopoietic stem cells and are known to play an important role in BBB maturation and stabilization (Armulik et al., 2010; Daneman et al., 2010). The human brain endothelial cell line hCMEC/D3 was first published in 2005 and has since been used in numerous studies on various aspects of BBB biology (Weksler et al., 2005; Qi et al., 2023). Although this cell line exhibits only weak barrier properties, it expresses important endothelial and BBB markers as well as various transporter proteins (Balzer et al., 2022). In addition, it is frequently used as a reference cell line for evaluating *in vitro* models derived from stem cells (Al-Ahmad, 2017).

Using these two established human *in vitro* BBB models, we investigated the effects of anti-HER2-targeted therapies for the treatment of advanced HER2-positive breast cancer to examine their influence on BBB integrity and possible consequences for cerebral metastases in HER2-positive breast cancer. We demonstrate that treatment of BLECs and hCMEC/D3 with lapatinib and tucatinib resulted in concentration-dependent effects: low doses increased cell viability, while higher concentrations were toxic. Furthermore, trastuzumab and trastuzumab/pertuzumab altered the mRNA expression of key BBB markers, suggesting complex, time- and concentration-dependent effects of anti-HER2 therapies on the BBB.

## Materials and methods

### Chemicals

Trastuzumab (145,531 g/mol), pertuzumab (148,000 g/mol) and lapatinib (581.06 g/mol) were obtained from the clinical pharmacy of University Hospital Würzburg. These drugs were then diluted in cell culture medium to the appropriate working concentrations of 5,000 ng/mL, 50 ng/mL, 5 ng/mL, and 0.5 ng/mL to be used in experiments. Tucatinib (480.5 g/mol), a specific HER2 inhibitor (HY-16069, Hycultec), was first dissolved in dimethyl sulfoxide (DMSO) at a stock concentration of 125 mg/mL. Following dissolution, tucatinib was further diluted in cell culture medium to match the same working concentrations of 5,000 ng/mL, 50 ng/mL, 5 ng/mL, and 0.5 ng/mL. The final concentration of the vehicle (DMSO) in the cell culture medium was below 1%.

This concentration range was chosen to represent a broad range, as there is limited data available on the exact clinically relevant free plasma or brain concentration. For lapatinib, for example, a range of 500–3,500 ng/mL in plasma and 1.3–4.5 ng/mL in cerebrospinal fluid was reported (Gori et al., 2014). More precise data on plasma concentrations (80–1,000 ng/mL) were available for tucatinib, which also depend on liver function (Topletz-Erickson et al., 2022).

### Cell culture

HER-2 positive breast cancer cell line BT 474 (HTB-20, ATCC) was grown in MEM medium (Thermo Fisehr Scientific, 51200-038) supplemented with 10% fetal calf serum (FCS), L-glutamine and penicillin/streptomycin. Triple-negative breast cancer cell line HCC 1806 (CRL-2335, ATCC) was grown in RPMI medium (R7509-500ML, Sigma-Aldrich) supplemented with 10% FCS, L-glutamine and penicillin/streptomycin. CD34^+^ hematopoietic stem cells were isolated from umbilical cord blood, following established protocols (Cecchelli et al., 2014; Lyck et al., 2017; Curtaz et al., 2020). After isolation, these stem cells were expanded in culture to increase their numbers. The cells were grown in Endothelial Cell Basal Medium, supplemented with the Microvascular Endothelial Cell Growth Supplement Kit containing 5% FCS (PB-BH-100-9806, PB-SH-100-4099, PELOBiotech). The cultures were maintained on Matrigel-coated transwells to promote cell adhesion and growth. For the coating, Matrigel growth factor reduced basement membrane matrix (354230, Corning) was thawed on ice and diluted 1:48 with cell culture medium to a concentration of approximately 300 μg/mL. Transwells inserts (12 well, 0.4 µm pore size, 1.12 cm^2^, Corning) were coated with 500 µL of diluted Matrigel for 1 h at room temperature. 8 × 10^4^ of cells were seeded per insert. To differentiate the CD34^+^ cells into brain like endothelial cells (BLECs), they were co-cultured with brain pericytes for 5 days (Cecchelli et al., 2014; Curtaz et al., 2020).

Human immortalized brain microvascular endothelial cell line, hCMEC/D3 (SCC066, Merck Millipore) was grown in Endothelial Cell Basal Medium, supplemented with the Microvascular Endothelial Cell Growth Supplement Kit containing 5% FCS (PB-BH-100-9806, PB-SH-100-4099, PELOBiotech) on gelatin or collagen I- coated plates and transwells.

### Immunocytochemistry

The cells were washed once with PBS and fixed for 15 min at room temperature with 2% formaldehyde. After two PBS washes, the cells were permeabilized with 0.1% Triton X-100 (Sigma-Aldrich). Cells were then washed twice with PBS and blocked for 1 h at room temperature in 5% normal swine serum blocking solution (S-4000, Vector Laboratories). The cells were then incubated overnight at 4 °C with a primary mouse monoclonal anti-ZO-1 (Zonula Occludens-1) antibody conjugated with Alexa Fluor™ 488 (33-9100, Thermo Fisher Scientific) at a 1:500 dilution in blocking solution. After three PBS washes, the cells were mounted with ProLong™ Gold Antifade Mountant (Themo Fisher Scientific) containing DAPI. The images were acquired using a fluorescence microscope (Axio Imager M2, Carl Zeiss AG) with a ×40 objective.

### Transendothelial electrical resistance and paracellular permeability measurements

BLECs (8 × 10^4^) were cultured in co-culture with pericytes on Matrigel-coated transwells as described above for 5 days. 8 × 10^4^ hCMEC/D3 were seeded on collagen I coated transwells (12 well, 0.4 µm pore size, 1.12 cm^2^, Corning) and grown to confluence for 3 days. After the cells reached confluence, they were left untreated or were treated with chemotherapies for 24 or 48 h. Following the treatment, transendothelial electrical resistance (TEER) was measured using manual voltohmmeter EVOM™ with chopstick electrode or ENDOHM-12 electrode (World Precision Instruments). To ensure a stable temperature during the measurement, the plates were held on a heating plate preheated to 37 °C. To calculate the TEER (Ohm x cm^2^), the measured resistance minus the resistance of a blank transwell covered by cell culture medium was multiplied by the surface area of the transwell (1.12 cm^2^) and normalized to untreated control. Subsequently, the transwell inserts were used to measure the paracellular permeability for the small tracer fluorescein (400 Da, F6377, Sigma-Aldrich). For the permeability measurement, the medium was replaced with pre-warmed HEPES-buffered Ringer’s solution (140 mM NaCl, 5 mM KCl, 2 mM CaCl_2_, 1 mM MgCl_2_, 25 mM NaHCO_3_, 5.5 mM HEPES, 1 mM D-glucose, pH 7.4). Inserts containing cells and control inserts without cells were placed in a 12-well plate containing 1.5 mL buffer solution. Subsequently, 0.5 mL of a 1 µM fluorescein solution in pre-warmed HEPES-buffered Ringer’s solution was added to the insert. The assay was performed for 1 hour using three filters per compound, with the inserts being transferred to a new well containing 1.5 mL of buffer every 20 min. All samples (stock solution, aliquots from the upper and lower compartments) and dilution series of fluorescein for the standard curve were measured at wavelengths 490/516 nm using a microplate reader (Tecan). The endothelial permeability coefficient (Pe), expressed in centimeters per minute was calculated using Excel (Microsoft).

### Cell viability assay

Cell viability assay with breast cancer cell lines BT474 and HC1806 and with endothelial cells was performed using a Cell Counting Kit-8 (E-CK-A362 Elabscience) according to the manufacturer’s instructions. Due to the different growth rates, the optimal seeding density was determined separately for each cell type. The cancer cell lines BT474 and HC1806 were seeded at a density of 5,000 cells per well in a 96-well plate and allowed to grow for 24 h before being treated with chemotherapies for 24 and 48 h. BLECs were seeded at a density of 1.1 × 10^4^ cells per well on gelatin-coated 96-well plates in pericyte-conditioned medium (fresh medium mixed with 20% filtered medium from a 24-h pericyte culture) and allowed to grow for 24 h before being treated with chemotherapies for 24 and 48 h. Endothelial cell line hCMEC/D3 was seeded at a density of 3,000 cells per well in collagen I-coated 96-well plates and allowed to grow for 24 h before being treated with chemotherapies for 24 and 48 h. Treatment with toxic concentrations of DMSO (10%) (D2650, Sigma Aldrich) was used as a positive control. The absorbance was measured at 450/600 nm using a microplate reader (Tecan).

### Real-time qPCR

Real-time quantitative polymerase chain reaction (qPCR) was carried out to assess gene expression levels, adhering to established protocols (Curtaz et al., 2022b; Feldheim et al., 2022). Total RNA was isolated using the NucleoSpin® RNA isolation kit (Macherey-Nagel) according to manufacturer’s instructions. For each condition, 1 µg of RNA was used as the starting material for complementary DNA (cDNA) synthesis. The RNA was converted to cDNA using the High-Capacity cDNA Reverse Transcription Kit (Thermo Fisher Scientific, 4368813). The qPCR reaction was prepared using the TaqMan® Fast Advanced Master Mix (Thermo Fisher Scientific, 4444965). Specific gene expression was quantified using TaqMan® Gene Expression Assays (Thermo Fisher Scientific, 4331182) designed and validated for the following target genes: ABCB1 (Hs00184500_m1), ABCG2 (Hs01053790_m1), CCL2 (Hs00234140_m1), CLDN5 (Hs00533949_s1), EGFR (Hs01076090), ERBB2 (Hs01001580), OCLN (Hs00170162_m1), SLC2A1 (Hs00892681_m1). CANX (calnexin, Hs01558409_m1) was used as an endogenous control. The qPCR reactions were run using the QuantStudio 7 Flex System (Thermo Fisher Scientific). After completion of the qPCR, the data were analyzed using QuantStudio™ Real-Time PCR Software v1.7.1 (Thermo Fisher Scientific).

### Statistical analysis

The statistical analysis was performed using GraphPad Prism 10 (GraphPad Software Inc.). Data are presented as the mean of three independent experiments with standard deviation. To determine statistical significance, Student’s t-test was used to compare differences between two groups. One-way ANOVA with Dunnetts’s multiple comparisons test or two-way ANOVA with Sidak’s multiple comparisons test was used to compare differences between three or more groups. P values below 0.05 were considered statistically significant and are marked as follows: *p < 0.05, **p < 0.01, ***p < 0.001, ****p < 0.0001.

## Results

### Characterization of chosen *in vitro* models and cell lines

**FIGURE 1:**
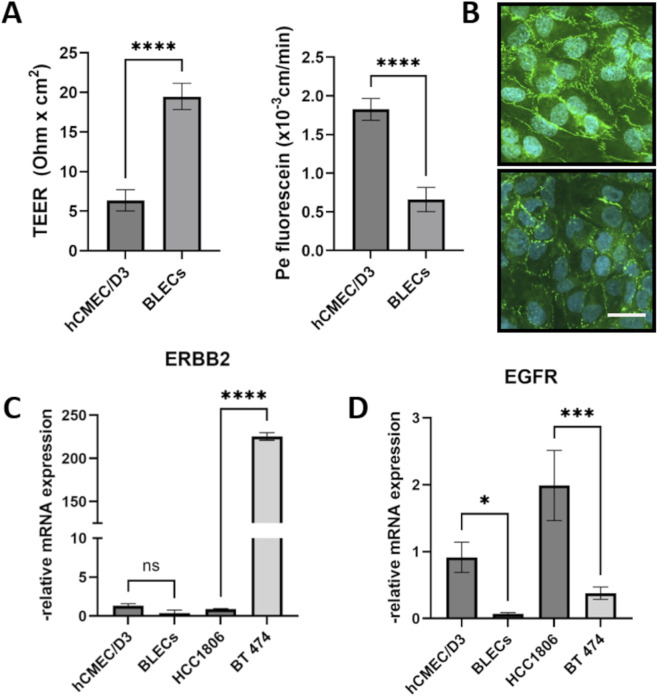
Characterization of selected *in vitro* models and cell lines. The transendothelial electrical resistance (TEER) and permeability coefficient (Pe) for fluorescein were measured in the immortalized endothelial cell line hCMEC/D3 and in CD34+-derived brain-like endothelial cells (BLECs) **(A)**. Subsequently, the tight junctions were immunostained with an anti-Zonlula Occludens (ZO)-1 antibody. Top image: BLECs, bottom image: hCMEC/D3, scale bar 20 µm **(B)**. Messenger RNA expression levels of HER2 (encoded by the ERBB2 gene) **(C)** and epidermal growth factor receptor (EGFR) **(D)** in brain microvascular endothelial cell line hCMEC/D3 and BLECs, as well as in the breast cancer cell lines HCC1806 and BT 474 were measured by qPCR. Data are presented as means ± standard deviation (n = 3). ns, not significant, *p < 0.05, ***p < 0.001, ****p < 0.0001.

To analyze the effects of HER2-targeted chemotherapies, we selected two established human BBB *in vitro* models, BLECs and hCMEC/D3. While BLECs are primary cells that can only be cultured for a limited number of passages, hCMEC/D3 is an immortalized cell line with a long culture duration. As shown in Figure 1A, BLECs exhibit higher barrier properties than hCMEC/D3, showing higher TEER values and lower paracellular permeability to the small tracer fluorescein. Both *in vitro* models form tight junctions, as demonstrated by immunofluorescence staining of the tight junction protein ZO-1 (Figure 1B). We then analyzed the expression of epidermal growth factor receptors 1 and 2 (EGFR, HER2) in BLECs, hCMEC/D3 and two breast cancer cell lines: BT 474 (HER2-positive breast cancer cell line) and HC 1806 (triple-negative breast cancer cell line) (Figures 1C,D). EGFR and HER2 expression levels are important indicators for HER2-targeted therapies. The results confirmed the established receptor status of these cells and showed a more than 200-fold overexpression of ERBB2 (HER2) in BT 474. EGF receptor expression levels were significantly lower in BT 474 than in HCC 1806. Analysis of ERBB2 and EGFR in brain microvascular endothelial cells, BLECs and hCMEC/D3 showed low ERBB2 and EGFR levels in both *in vitro* models (Figures 1C,D). Interestingly, the hCMEC/D3 cell line expressed significantly higher EGFR levels than BLECs.

### Effects of anti-HER2-targeted therapies on cell viability

**FIGURE 2:**
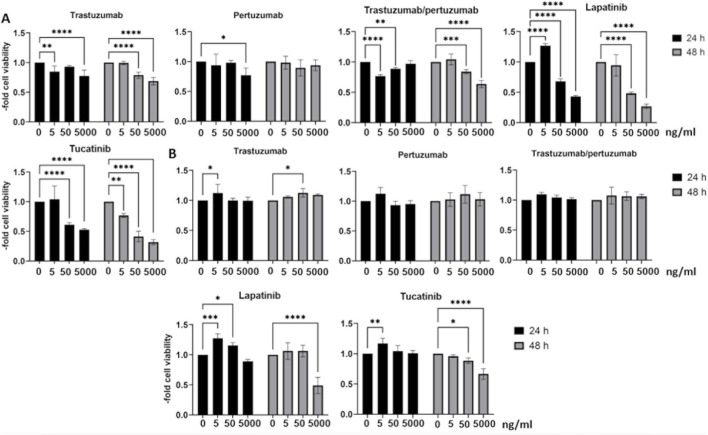
Cell viability in breast cancer cell lines. The HER2-positive BT 474 cell line **(A)** and the triple-negative HC1806 breast cancer cell lines **(B)** were treated with 5, 50, 5,000 ng/mL trastuzumab, pertuzumab, trastuzumab/pertuzumab, lapatinib and tucatinib for 24 or 48 h followed by cell viability assay. Data are presented as means ± standard deviation and expressed as the fold of the untreated control, which was set at 1 (n = 3). *p < 0.05, **p < 0.01, ***p < 0.001, ****p < 0.0001 compared to the untreated control.

We selected a broad concentration range of chemotherapies and first investigated their cytotoxic effects in breast cancer cell lines with different receptor statuses. After 24 or 48 h of treatment with HER2-targeted chemotherapies, cell viability was measured in the HER2-positive BT 474 breast cancer cell line (Figure 2A) and the triple-negative breast cancer cell line HC 1806 (Figure 2B). Almost all HER2-targeted chemotherapies showed toxic effects on HER2-overexpxressing BT474 cells; only pertuzumab showed no or only low toxicity at the highest concentration (Figure 2A). While treatment with trastuzumab, pertuzumab and their combination was not toxic to the HER2-negative breast cancer cell line HC1806 after 24 or 48 h, lapatinib and tucatinib showed toxic effects after 48 h of treatment at a concentration of 5,000 ng/mL.

**FIGURE 3:**
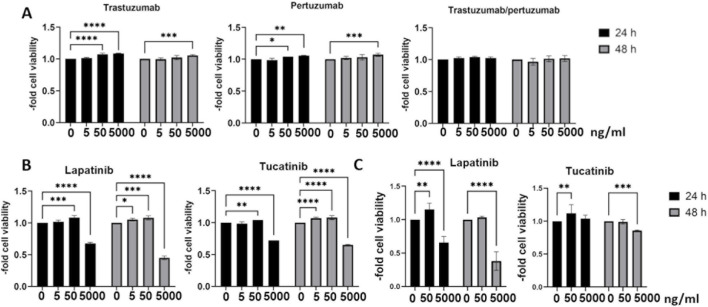
Cell viability in human blood-brain barrier *in vitro* models. Cell viability was measured after 24 h or 48 h treatment with trastuzumab, pertuzumab, trastuzumab/pertuzumab **(A)**, lapatinib and tucatinib **(B)** in BLECs and in hCMEC/D3 **(C)** after treatment with lapatinib and tucatinib. Data are presented as means ± standard deviation and expressed as the fold of the untreated control, which was set at 1 (n = 3). *p < 0.05, **p < 0.01, ****p < 0.0001 compared to the untreated control.

Next, we tested the same concentrations of chemotherapies in brain endothelial cells (Figure 3). Treatment with 5, 50 or 5,000 ng/mL trastuzumab, pertuzumab or trastuzumab/pertuzumab for 24 h and 48 h showed no toxic effects in BLECs (Figure 3A). The concentration of 5,000 ng/mL lapatinib and tucatinib was toxic to BLECs after 24 and 48 h of treatment (Figure 3B). We have chosen therefore lapatinib and tucatinib to treat the second *in vitro* BBB model, hCMEC/D3 (Figure 3C). Lapatinib and tucatinib showed toxic effects in higher concentrations and longer treatment times also in these cells. Therefore, in subsequent experiments, we used chemotherapies only in low, non-toxic concentrations of 0.5, 5 and 50 ng/mL for BLECs with a treatment duration of 24 h and 50 ng/mL for hCMEC/D3 with a treatment duration of 24 and 48 h.

### Effects of HER2-targeted therapies on the barrier properties of *in vitro* BBB models

**FIGURE 4:**
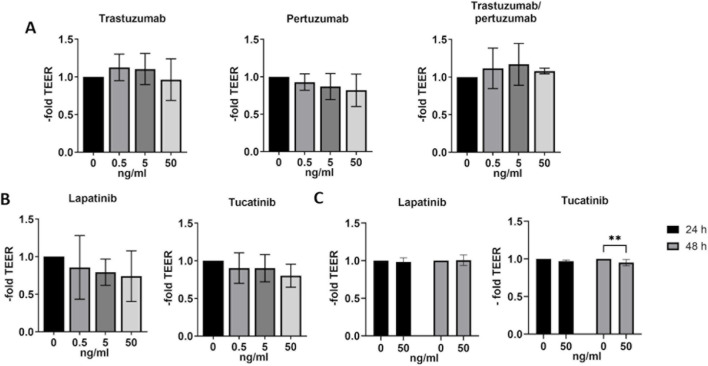
Transendothelial electrical resistance (TEER) in human blood-brain barrier *in vitro* models. TEER was measured after 24 h treatment with trastuzumab, pertuzumab, trastuzumab/pertuzumab **(A)**, lapatinib and tucatinib **(B)** in BLECs and in hCMEC/D3 **(C)** after treatment with lapatinib and tucatinib for 24 h and 48 h. Data are presented as means ± standard deviation and expressed as the fold of the untreated control, which was set at 1 (n = 3). **p < 0.01 compared to the untreated control.

Measurements of barrier properties such as TEER and paracellular permeability are well established methods for assessing the functional effects of various substances, molecules or conditions on the BBB *in vitro* models. BLECs were treated with 0.5, 5 or 50 ng/mL of trastuzumab, pertuzumab, trastuzumab/pertuzumab (Figure 4A), lapatinib or tucatinib (Figure 4B) for 24 h and hCMEC/D3 were treated with 50 ng/mL lapatinib or tucatinib for 24 h and 48 h (Figure 4C). The changes in TEER levels were normalized to the respective untreated control. While no significant changes in TEER were found in BLECs (Figures 4A,B), hCMEC/D3 showed a slight difference in TEER after 48 h treatment with 50 ng/mL of tucatinib.

**FIGURE 5:**
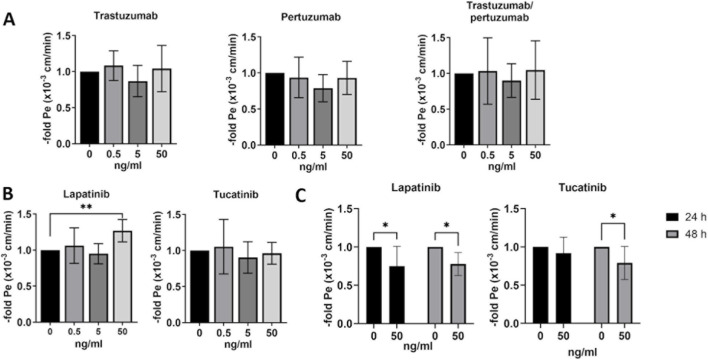
Paracellular permeability in human blood-brain barrier *in vitro* models. Paracellular fluorescein permeability was measured and permeability coefficient was calculated after 24 h of treatment with trastuzumab, pertuzumab, trastuzumab/pertuzumab **(A)**, lapatinib and tucatinib **(B)** in BLECs and in hCMEC/D3 after treatment with lapatinib and tucatinib for 24 h and 48 h **(C)**. Permeability coefficient is presented as means ± standard deviation and expressed as the fold of the untreated control, which was set at 1 (n = 3). *p < 0.05, **p < 0.01 compared to the untreated control.

BLECs and hCMEC/D3 were treated with chemotherapies as described above, followed by 1-h permeability assay using a fluorescein. The permeability coefficient was calculated for each condition and expressed as fold of the untreated control, which was set to 1 (Figure 5). Trastuzumab, pertuzumab and their combination showed no effects on endothelial cell permeability in BLECs (Figure 5A). Lapatinib at a concentration of 50 ng/mL significantly increased the permeability of BLECs after 24 h (Figure 5B). While 50 ng/mL lapatinib resulted in reduced permeability in hCMEC/D3 after 24 and 48 h of treatment, 50 ng/mL tucatinib reduced permeability only after 48 h of treatment. Lapatinib and tucatinib may therefore have a barrier-modulating effect in both hCMEC/D3 and BLECs, however, the lack of effects on TEER requires further investigation with additional *in vitro* models.

### Changes in mRNA expression in BLECs after treatment with anti-HER2-targeted chemotherapies

**FIGURE 6:**
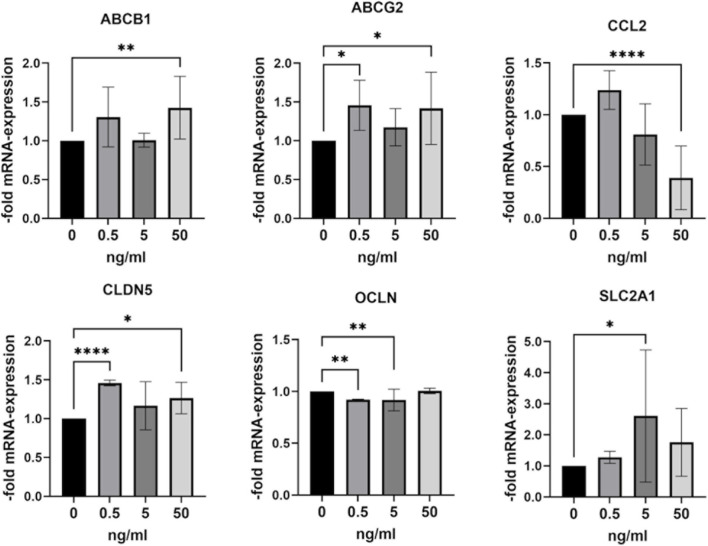
Treatment of BLECs with trastuzumab/pertuzumab leads to changes in the mRNA expression of BBB markers. BLECs were treated with 0.5, 5 or 50 ng/mL trastuzumab/pertuzumab for 24 h or left untreated. Subsequently, gene expression analysis was performed using qPCR. Data are presented as means ± standard deviation of fold mRNA expression over the untreated control, which was set at 1 (n = 3). ABCB1: ATP Binding Cassette Subfamily B Member 1; ABCG2: ATP Binding Cassette Subfamily G Member 2; CCL2: C-C Motif Chemokine Ligand 2; CLDN5: claudin-5; OCLN: occludin, SLC2A1: Solute Carrier Family 2 Member 1. *p < 0.05, **p < 0.01, ****p < 0.0001 compared to the untreated control.

In the next step, we investigated the effects of chemotherapies on the mRNA expression of endothelial and BBB marker genes in BLECs (Figure 6 and Supplementary Table S1). BLECs were treated with 0.5, 5 or 50 ng/mL of trastuzumab, pertuzumab, trastuzumab/pertuzumab, lapatinib and tucatinib for 24 h and then examined for mRNA expression using qPCR. As shown in Figure 6, the expression of the efflux pumps ATP Binding Cassette Subfamily B Member 1 (ABCB1) and ATP Binding Cassette Subfamily G Member 2 (ABCG2) was significantly increased after treatment with 50 ng/mL trastuzumab/pertuzumab. In addition, trastuzumab/pertuzumab significantly reduced the mRNA expression of the proinflammatory cytokine C-C Motif Chemokine Ligand 2 (CCL2) at concentrations of 0.5 ng/mL and 50 ng/mL, respectively. While the mRNA expression of the tight junction protein claudin-5 (CLDN5) was significantly increased after treatment with 0.5 and 50 ng/mL trastuzumab/pertuzumab, the mRNA expression of another tight junction protein, occludin (OCLN), was significantly decreased after treatment with 0.5 and 5 ng/mL trastuzumab/pertuzumab. The mRNA expression of glucose transporter 1, encoded by the Solute Carrier Family 2 Member 1 (SLC2A1) gene, was significantly increased after treatment with 5 ng/mL trastuzumab/pertuzumab. In addition to the combination of trastuzumab/perutuzumab, trastuzumab alone led to increased mRNA expression of ABCB1 and ABCG2 at a concentration of 50 ng/mL, a decrease in CCL2 mRNA expression at 0.5 ng/mL, and a significant increase in mRNA expression of CLDN5, OCLN and SLC2A1 at a concentration of 50 ng/mL (Supplementary Table S1). Pertuzumab alone led to a significant increase of SLC2A1 at a concentration of 0.5 ng/mL (Supplementary Table S1). Treatment with the two TKIs, lapatinib and tucatinib did not result in any significant change in gene expression (Supplementary Table S1).

## Discussion

Here, we analyzed the effects of clinically used chemotherapy in HER2-positive breast cancer on the BBB *in vitro* models, since patients with HER2-positive breast cancer had the highest rate of brain metastases after patients with triple-negative breast cancer, but also the best survival data (Müller et al., 2025). Furthermore, a study that analyzed HER2 expression in resected human brain metastases showed an increase in the frequency of HER2 overexpression in brain metastases (Palmieri et al., 2007). In recent years, chemotherapy for HER2-positive breast cancer has developed rapidly. In addition to anti-HER2 antibodies (trastuzumab, pertuzumab), TKIs (lapatinib, tucatinib, neratinib), antibody-drug conjugates (ADC) such as trastuzumab/emtansine or trastuzumab/deruxtecan are currently used and recommended (Müller et al., 2025). Metastatic tumor cells and chemotherapy for brain metastases must overcome the BBB on their way to the brain. On the other hand, chemotherapies used to treat breast cancer can affect the properties of the BBB and these effects are largely unknown (Curtaz et al., 2022a). Using HER2-positive and -negative breast cancer cell lines, we confirmed selective toxicity of the chemotherapies on the HER2-positive line. We demonstrate an initial proof-of-concept with BT474 (HER2+/ER+/PR+) and HCC 1806 (HER2-/triple-negative) as representative luminal and basal-like models. Although HCC1806 is of squamous origin, it is widely accepted as a triple-negative model. Due to time and resource constraints, additional subtypes (e.g., HER2+/ER−/PR−, HER2−/ER+/PR+) were not included. We chose 24 h and 48 h to capture early signaling prior to cytotoxicity, and emphasized that longer treatment durations might capture adaptive rather than acute BBB responses. Future studies should therefore include more models and longer exposure times to strengthen generalizability. Despite their mutations and their inability to fully recapitulate tumors, breast cancer cell lines remain valuable models for breast cancer subtypes (Dai et al., 2017). This supports our use of selected cell lines in this preliminary study and shows how important it is to expand the model system in future work.

Many chemotherapy drugs, including trastuzumab and pertuzumab, cross the BBB only poorly, with cerebrospinal fluid levels of trastuzumab about 300-fold lower than plasma (Venur and Leone, 2016). Although radiolabeled trastuzumab could be detected in brain metastases (Tamura et al., 2013), the efficacy of trastuzumab and pertuzumab in the treatment of brain metastases is limited. In the event of a disruption of the BBB, higher doses of trastuzumab and pertuzumab can reach the brain (Lin et al., 2021). In contrast, TKIs such as lapatinib and tucatinib with lower molecular weight seem to penetrate the BBB more efficiently showing promising treatment option for HER2-positive breast cancer with brain metastases (Duchnowska et al., 2018; Nader-Marta et al., 2022; Wang et al., 2022; Werter et al., 2022). The exact efficacy and molecular absorption of these substances remain poorly understood, as only few *in vivo* and *in vitro* models exist and data on their effects on BBB permeability are particularly rare (Terrell-Hall et al., 2017; Cordero et al., 2022; Li et al., 2022). In the current national and international guidelines for the treatment of HER2-positive breast cancer with brain metastases, the TKI tucatinib and the ADC trastuzumab-deruxtecan are currently recommended as first line therapy for systemic therapy regimes (Le Rhun et al., 2021; Vogelbaum et al., 2022; Müller et al., 2025). The most recently approved ADC trastuzumab-deruxtecan was not used in this study because it was not yet approved for the treatment of cerebral metastasis at the time of the experimental work (Bartsch et al., 2022). Further studies with these substances are therefore still pending.

While studies on the BBB in breast cancer brain metastases are still limited, findings from other cancers with cerebral dissemination (e.g., lung cancer, melanoma) and primary brain tumors (glioblastoma) are already available (Mo et al., 2021).

In our study, after treatment with lapatinib and tucatinib, cell viability assays demonstrated increased endothelial viability at lower concentrations, while high concentrations were toxic to BLECs and hCMEC/D3. Since these results were observed with both TKI drugs, coincidence is highly unlikely. This can be interpreted that only a specific concentration range of TKI, namely low concentrations, have a positive effect on cell viability and elicit an adaptive response, while higher concentrations show a directly toxic effect. The cell viability assay is often used to measure cellular metabolic activity as an indicator for cell viability, proliferation and cytotoxicity (Präbst et al., 2017). Tyrosine kinases phosphorylate particular amino acids of substrate enzymes, which then modify signal transduction, leading to subsequent changes in cell biology. The use of TKIs lapatinib and tucatinib at high concentrations may affect the integrity of the endothelial cell barrier, while at low concentrations they are more likely to support the endothelial metabolic activity and barrier properties as observed in BLECs and hCMEC/D3. This could be an effect of the adaptive cellular response to chemotherapeutic stress on endothelial cells, which is more pronounced with longer treatment durations. A structural analog of lapatinib, GW2974, resulted in a significant increase in paracellular permeability to fluorescein and FITC-avidin (Curtaz et al., 2024). GW2974 has similar pharmacological properties as lapatinib (Sodani et al., 2012), but was not advanced into clinical trials due to pharmacokinetic issues. Therefore, the different substances within the TKI substance group can differ in their effect on the BBB depending on the concentration and the time of treatment. Further studies on these effects are needed to validate these effects. The absence of cytotoxic effects of trastuzumab and pertuzumab as well as their combination on endothelial cell viability, even at the highest concentrations, could be attributed to the different mechanisms of action of these chemotherapies. While TKIs inhibit the intracellular domain of HER2 and/or EGFR, anti-HER2 antibodies bind to the extracellular domain of HER2. The mRNA expression levels of HER2, encoded by ERBB2 gene, showed that both *in vitro* BBB models, BLECs and hCMEC/D3 cells exhibit low HER2 mRNA levels and differ significantly in EGFR expression, with higher mRNA expression levels in hCMEC/D3 cells. This could explain the stronger effects of lapatinib and tucatinib on hCMEC/D3 compared to BLECs observed in fluorescein permeability measurements. However, since no effects on TEER were observed, further investigations with additional *in vitro* models are required.

EGFR signaling is involved in the regulation of gene expression in endothelial cells, e.g. it has been shown that the tight junction protein occludin in brain endothelial cells of mice with acute liver failure was regulated by EGFR (Chen et al., 2011). To assess changes in gene expression in BLECs, the efflux pumps PG-P (ABCB1) and BCRP (ABCG2), the glucose transporter GLUT-1 (SLC2A1), the tight junction proteins occludin (OCLN) and claudin-5 (CLDN5), and the proinflammatory chemokine CCL2 were examined. The ABCB1/ABCG2 efflux system at the BBB poses a significant problem for successful drug delivery to the brain, as it hinders the uptake of anti-cancer drugs into the brain and severely limits their efficacy in treating primary and metastatic brain tumors (Schulz et al., 2023). Anti-HER2 antibodies trastuzumab and pertuzumab cannot effectively cross the BBB due to their size. The binding of trastuzumab to the neonatal Fc receptor (FcRn), which is also expressed in brain endothelial cells and the hCMEC/D3 cell line (Urich et al., 2012), could influence the effects of the antibodies in these cells. BLECs expressed significantly higher levels of the efflux pumps ABCB1 and ABCG2 mRNA after treatment with trastuzumab and trastuzumab/pertuzumab. In addition, the mRNA levels of the genes encoding the tight junction proteins CLDN5 and OCLN showed altered expression patterns after treatment with trastuzumab and trastuzumab/pertuzumab. These two tight junction proteins are considered to be the most important proteins that form the main pillar of the tight junctions and crucially determine the paracellular tightness between neighboring endothelial cells of the BBB (Lochhead et al., 2020) and further analyses at the protein level are required. GLUT1 has a high expression level in endothelial cells in the central nervous system, as it is a main glucose transporter in endothelial cells. GLUT1 is often associated with disorders of the BBB (Zheng et al., 2010; Veys et al., 2020). The mRNA expression of SLC2A1 increased significantly after treatment with trastuzumab, pertuzumab, and trastuzumab/pertuzumab at selected concentrations. Neither lapatinib nor tucatinib affected gene expression in BLECs at the selected concentrations and treatment duration.

Our study has limitations, as only a limited number of experimental conditions could be tested. Longer treatment durations and standardized testing of individual drugs across multiple *in vitro* models are needed to better reflect clinical conditions. As previously shown, factors from the serum of breast cancer patients can influence BBB properties (Curtaz et al., 2020; Curtaz et al., 2022b; Teles et al., 2025), and this can also be assumed for breast cancer cells. Therefore, co-culture with breast cancer cells and the investigation of cancer cell adhesion and diapedesis could also be useful for such experiments.

## Data Availability

The original contributions presented in the study are included in the article/Supplementary Material, further inquiries can be directed to the corresponding author.
