# Supplementary material for: Anti-HER2-targeted therapies: effects on human in vitro blood-brain barrier models
Source: Front Drug Deliv. 2026 Jan 7;5:1700455. doi: 10.3389/fddev.2025.1700455 (PMC12819789; doi:10.3389/fddev.2025.1700455)
Supplement: Supplementary file 1 [file Table1.docx]

**Supplemental Table 1.** mRNA expression levels in BLECs after treatment with chemotherapies for HER2-positive breast cancer.

| **Gene**  **Treatment** | | **ABCB1** | **ABCG2** | **CCL2** | **CLDN5** | **OCLN** | **SLC2A1** |
| --- | --- | --- | --- | --- | --- | --- | --- |
| Trastuzumab | 50 ng/ml | 3.2 ± 3.04* | 4.2 ± 4.66* | 0.9 ± 0.20 | 3.1 ± 2.37* | 1.9 ± 1.46* | 1.9 ± 0.92** |
|  | 5 ng/ml | 1.6 ± 0.47 | 1.7 ± 1.21 | 0.8 ± 0.54 | 1.4 ± 0.44 | 1.3 ± 0.31 | 1.4 ± 0.13 |
|  | 0.5 ng/ml | 1.3 ± 0.33 | 1.3 ± 0.49 | 0.6 ± 0.35* | 1.5 ± 0.45 | 1.2 ± 0.28 | 1.1 ± 0.07 |
| Pertuzumab | 50 ng/ml | 1.3 ± 0.48 | 1.4 ± 0.68 | 1.0 ± 0.64 | 1.2 ± 0.39 | 1.3 ± 0.44 | 1.3 ± 0.37 |
|  | 5 ng/ml | 1.5 ± 0.72 | 1.8 ± 1.44 | 1.1 ± 0.52 | 1.5 ± 0.98 | 1.4 ± 0.42 | 1.5 ± 0.67 |
|  | 0.5 ng/ml | 1.4 ± 0.50 | 1.6 ± 0.90 | 1.3 ± 0.39 | 1.3 ± 0.69 | 1.3 ± 0.52 | 2.0 ± 1.16* |
| Lapatinib | 50 ng/ml | 1.0 ± 0.13 | 1.1 ± 0.14 | 1.0 ± 0.20 | 1.0 ± 0.19 | 1.0 ± 0.13 | 1.1 ± 0.11 |
|  | 5 ng/ml | 0.9 ± 0.05 | 0.9 ± 0.21 | 1.1 ± 0.31 | 1.1 ± 0.09 | 1.0 ± 0.09 | 1.1 ± 0.16 |
|  | 0.5 ng/ml | 1.1 ± 0.04 | 1.0 ± 0.21 | 1.1 ± 0.39 | 1.0 ± 0.19 | 1.0 ± 0.17 | 1.0 ± 0.11 |
| Tucatinib | 50 ng/ml | 0.8 ± 0.29 | 0.9 ± 0.20 | 0.7 ± 0.30 | 0.7 ± 0.33 | 0.9 ± 0.37 | 0.9 ± 0.06 |
|  | 5 ng/ml | 0.6 ± 0.33 | 0.9 ± 0.29 | 0.7 ± 0.25 | 0.8 ± 0.46 | 0.7 ± 0.35 | 1.0 ± 0.14 |
|  | 0.5 ng/ml | 0.9 ± 0.07 | 1.1 ± 0.21 | 0.9 ± 0.09 | 1.1 ± 0.10 | 1.1 ± 0.17 | 1.0 ± 0.02 |

Data are presented as means ± standard deviation of fold mRNA expression over the untreated control, which was set at 1 (n = 3). ABCB1: ATP Binding Cassette Subfamily B Member 1; ABCG2: ATP Binding Cassette Subfamily G Member 2; CCL2: C-C Motif Chemokine Ligand 2; CLDN5: claudin-5; OCLN: occludin, SLC2A1: Solute Carrier Family 2 Member 1. * p < 0.05, ** p < 0.01 compared to the untreated control.
